# Shuffle Attention-Based Pavement-Sealed Crack Distress Detection

**DOI:** 10.3390/s24175757

**Published:** 2024-09-04

**Authors:** Bo Yuan, Zhaoyun Sun, Lili Pei, Wei Li, Kaiyue Zhao

**Affiliations:** 1 School of Data Science and Artificial Intelligence, Chang’an University, Xi’an 710061, China; yuanbo_chd@chd.edu.cn (B.Y.); peilili@chd.edu.cn (L.P.); grandy@chd.edu.cn (W.L.); 2 School of Information Engineering, Chang’an University, Xi’an 710064, China; 2021124030@chd.edu.cn

**Keywords:** neural network, distress detection, pavement-sealed crack, shuffle attention, wise intersection over union

## Abstract

To enhance the detection of pavement-sealed cracks and ensure the long-term stability of pavement performance, a novel approach called the shuffle attention-based pavement-sealed crack detection is proposed. This method consists of three essential components: the feature extraction network, the detection head, and the Wise Intersection over Union loss function. Within both the feature extraction network and the detection head, the shuffle attention module is integrated to capture the high-dimensional semantic information of pavement-sealed cracks by combining spatial and channel attention in parallel. The two-way detection head with multi-scale feature fusion efficiently combines contextual information for pavement-sealed crack detection. Additionally, the Wise Intersection over Union loss function dynamically adjusts the gradient gain, enhancing the accuracy of bounding box fitting and coverage area. Experimental results highlight the superiority of our proposed method, with higher mAP@0.5 (98.02%), Recall (0.9768), and F1-score (0.9680) values compared to the one-stage state-of-the-art methods, showcasing improvements of 0.81%, 1.8%, and 2.79%, respectively.

## 1. Introduction

Pavement-sealed cracks encompass the procedure of repairing and preventing the enlargement or formation of cracks in pavement structures by filling them with designated materials. The regular detection of these cracks is essential for averting structural damage, improving safety, and prolonging their lifespan [1,2]. However, manual detection methods often suffer from subjective judgments, resulting in inconsistent detection standards and low efficiency.

Advancements in computer vision technology have led to the emergence of an automated approach for pavement defect detection, utilizing image processing techniques [3,4,5]. Two main categories of methods are used for pavement crack detection: traditional mathematical methods based on thresholds and deep learning methods. Sun et al. [6] introduced a method that focuses on the extraction and connection of pavement crack breakpoints. This approach involves tracing the direction of crack pixels to identify breakpoints and connecting them to the nearest crack point. Jing et al. [7] devised a method to tackle the distinctive features of cracks in pavement images. They utilized bilinear interpolation to create corrected images by analyzing the signal model and extracting background subsets from the original pavement images. The segmentation threshold was subsequently determined through statistical guidelines by adjusting the histogram of the image. Salari et al. [8] utilized a digital camera to capture pavement images, detected crack areas through local segmentation, and represented them as matrices of blocks. By calculating the standard deviation of vertical and horizontal histograms, cracks were mapped onto a two-dimensional feature space to classify them into four types: longitudinal, transverse, block, and alligator cracks. Abbas et al. [9] proposed an automated method for pavement disease detection, utilizing image processing technology to identify various types of pavement distress. However, traditional mathematical methods employed in the aforementioned approaches are susceptible to image quality, low contrast in distress features, lighting shadows, and other factors, resulting in unstable performance in real-world engineering environments.

In recent years, advancements in deep learning for image recognition and detection have introduced novel perspectives for addressing pavement distresses. By harnessing extensive data learning, these methods achieve target recognition from high-dimensional space. This approach helps overcome the challenges posed by the variability in pavement distress characteristics, thereby mitigating the issue of low recognition rates. Du et al. [10] constructed a comprehensive dataset for pavement distresses and employed the You Only Look Once network (YOLO), a deep learning-based object detection framework, to predict the potential locations and categories of distresses. Additionally, the adaptability of the model in diverse lighting conditions was discussed. Zhang et al. [11] proposed and evaluated an automatic method for detecting and classifying pavement distress types using a convolutional neural network (CNN) and a cost-effective video data collection strategy. They built and tested a dataset of images collected from roadways in Montreal, leveraging a sensitivity analysis to evaluate various regularization scenarios and data generation strategies. Zhu et al. [12] proposed the utilization of Unmanned Aerial Vehicles (UAVs) equipped with high-resolution cameras to capture images of road surface distresses and perform detection. The evaluation encompassed three object detection algorithms: R-CNN, YOLOv3, and YOLOv4. Li et al. [13] introduced a transfer learning approach for addressing cross-scene pavement distress detection by utilizing data transmission and model transfer techniques. The data transfer training involves generating adversarial networks that convert existing image data into new scene styles. Additionally, CutMix and image fusion were employed to incorporate distress annotations and synthesize labeled data for new scenes. Subsequently, the extracted features from the pre-existing model were transferred to the detection application in the new scene through domain adaptation. Guan et al. [14] developed an automatic pavement distress detection system. Their framework fused stereo vision and deep learning techniques to enable the automatic pixel-level detection of pavement distresses. They proposed an enhanced U-net architecture by incorporating deep separable convolution for an accurate segmentation of cracks and potholes in 3D pavement images, achieving a high level of Precision at the millimeter scale. Yuan et al. [15] introduced an enhanced generative adversarial network-based super-resolution reconstruction method for pavement diseases. In this method, batch normalization was incorporated into the nonlinear network through the creation of residual dense blocks (RDBs). Additionally, an attention module is formed by combining RDB, gated recurrent unit, and convolutional layers. The original loss function is substituted with an L1 norm-based loss function. The authors further employed Faster-RCNN and a fully convolutional network to exhibit an enhanced detection and segmentation of reconstructed pavement diseases. Yuan et al. [16] proposed an airport concrete pavement joint detection network based on dual-modal feature fusion (ACJD-DFF). They proposed outlier cleaning and row-level distortion correction techniques to enhance the quality of 3D concrete pavement images and generate a dataset consisting of a dual-modal feature fusion matrix. ACJD-DFF accurately maps the positioning coordinates of the joints, allowing for the quantification of joint misalignment. Du et al. [17] proposed a modeling automatic pavement crack object detection and pixel-level segmentation method, which realized the location of cracks by modifying YOLOv4-Tiny, and a further attention feature pyramid network was proposed to compensate for the loss caused by the reduction in the backbone network. Based on the characteristics of crack images, Yang et al. [18] developed a dense redundant crack annotation method. Positioning is more accurate than traditional annotations. In order to achieve efficient crack detection, a semi-automatic crack marking method was proposed, which reduced the working time by 80% compared with full manual marking. Wang et al. [19] proposed an effective method for measuring crack length. The method consists of a detection module based on an object detection algorithm and a length calculation module. In order to improve the speed and accuracy of crack detection, they proposed an improved pavement crack detection algorithm, YOLO V5-BiFPN, based on YOLOV5 and Bidirectional Feature Pyramid Network (BiFPN).

Based on the above literature review, the development of deep learning has provided new technical support for pavement distress detection. However, there are still the following problems for pavement-sealed crack detection: (1) Although deep learning methods abstractly extract features through deep convolutional networks, they do not specifically differentiate between spatial and channel information. (2) Existing models utilize Intersection over Union (IoU) to filter out bounding boxes, but they fail to adequately penalize low-quality samples. To address the above issues, the shuffle attention-based pavement-sealed crack detection (PSD) is proposed with the following innovations:

(1) The shuffle attention (SA) module is integrated into both the feature extraction network and detection head. This module performs a differential analysis of spatial and channel information, allowing the network to precisely capture contextual information.
(2) To suppress the bounding box cover area inaccurately caused by low-quality samples, the Wise Intersection over Union (WIoU) loss function for dynamically adjusting the gradient gain is employed to improve the fitting effect of the bounding box.
(3) A customized multi-scale fused two-way detection head is introduced to improve the accuracy of pavement-sealed cracks.

## 2. Methodology

This section proposes the PSD model framework, which comprises three essential components: a feature extraction network, a detection head, and a WIoU loss function. The SA module is integrated into both the feature extraction network and the detection head, allowing for a differential analysis of spatial and channel information. To facilitate the detection of pavement-sealed cracks, a two-way detection head is utilized, enabling multi-scale feature fusion. In addition, the WIoU loss function is employed to dynamically adjust the gradient gain.

### 2.1. Feature Extraction Network

**Figure 1:**
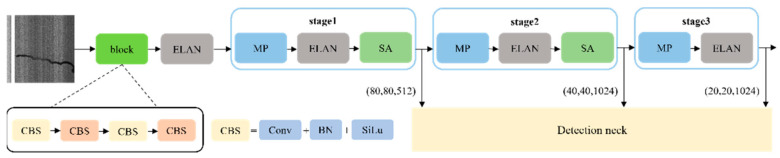
The structure of the feature extraction network.

A feature extraction network is utilized to extract detailed information and high-dimensional abstract features. The PSD receives grayscale pavement-sealed crack images with a resolution of 640 × 640. After the feature extraction network, three sets of feature maps at different scales are obtained. In order to maintain spatial pixel-level relationships and capture channel dependencies, the SA module is integrated after stages 1 and 2 of the Efficient Layer Aggregation Network (ELAN). The structure of the feature extraction network is illustrated in Figure 1. Finally, the feature extraction network will output three feature extraction maps with different scales and number of channels, in which the feature map with the output size of (80, 80) in Stage 1 contains 512 channels, and the feature map corresponding to (40, 40) and (80, 80) corresponding to 1024 channels in Stage 2 and Stage 3 are output, respectively.

**Figure 2:**
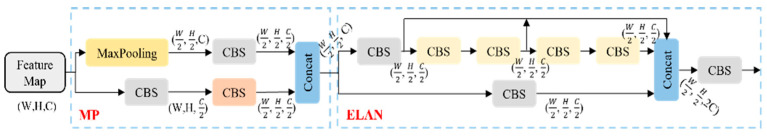
The structure of MP and ELAN.

To address the issue of detail loss caused by maximum pooling during downsampling, a Max Pooling (MP) module is constructed, as depicted in Figure 2. CBS is the basic unit of the model, which consists of Conv, batch normalization, and activation function; in this model, the activation function of CBS is SiLu. The MP module consists of two branches: one combines maximum pooling with a CBS, while the other cascades CBS with various convolution kernels and strides. By combining different downsampling methods, MP achieves the benefits of maximum pooling without sacrificing feature details. It introduces no additional computational burden and enhances the downsampling effect.

The ELAN module, also illustrated in Figure 2, consists of a main link and a multi-branch bypass connection. Within this module, different CBSs are cascaded and concatenated to expand the model’s receptive field. The ELAN module produces output features of the same size as the input, with the number of channels remaining the same or doubling.

The purpose of ELAN is to enhance the expressive ability of features and the propagation efficiency of gradients. In ELAN, W, H, and C represent the width, height, and number of channels of the feature map, respectively. To conform is to stitch the input feature map in the channel dimension. It consists of multiple branches processing feature maps in parallel and then aggregating them in cascading connections. ELAN helps networks learn richer and more multi-layered representations of features while maintaining computational efficiency.

**Figure 3:**
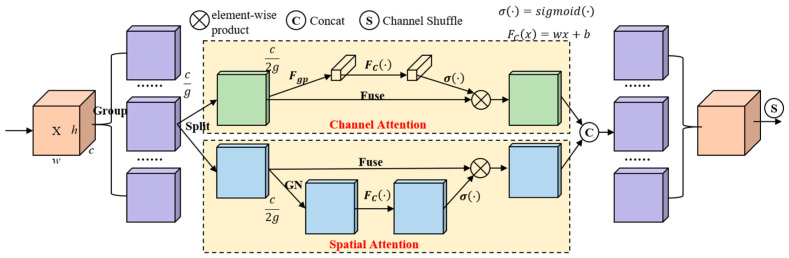
The structure of SA.

To handle the distinctive information within pavement-sealed cracks in terms of both spatial and channel dimensions, the SA module [20] is introduced. This module is divided into blocks to effectively employ spatial and channel attention mechanisms. Subsequently, channel shuffling is performed to integrate the obtained results. The structure of the SA module is illustrated in Figure 3.

The input feature map is first divided into g groups according to the channels, and the separated set of features ($\frac{c}{g}$) is split into two parts, ($\frac{c}{2g}$), which are sent to channel attention and spatial attention, respectively. In the picture “...” is the omission of multiple sets of separation results. The channel attention branch involves performing global average pooling ($F_{gp}$) on the input SA module, obtaining the channel weight value through fully connected layers ($F_{c}(\cdot )$) and a sigmoid activation function (σ(·)), and then performing element-wise with the input SA module to restore the feature map at the same scale. This process achieves channel position weighting. In the spatial attention branch, the input SA module undergoes Group Normalization (GN), followed by obtaining the attention weight for each pixel through $F_{C}$ and σ(·). The input SA module is multiplied by attention weights. The output of the SA module is obtained by channel shuffling the SA module after concatenating the results from two parallel computation branches.

The incorporation of the SA module enables the model to precisely focus on important features and reduce information redundancy, thereby enhancing the perception capability of the feature extraction network.

### 2.2. Detection Head for Sealed Crack

**Figure 4:**
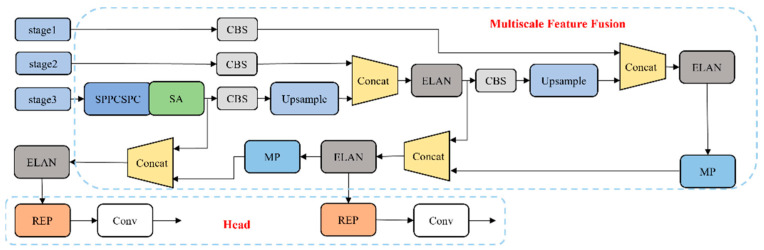
The structure of the detection head.

The detection head is utilized to identify pavement-sealed cracks of different sizes, and it transforms the output of the network into the position and confidence of the predicted bounding box. The structure of the detection head is illustrated in Figure 4.

**Figure 5:**
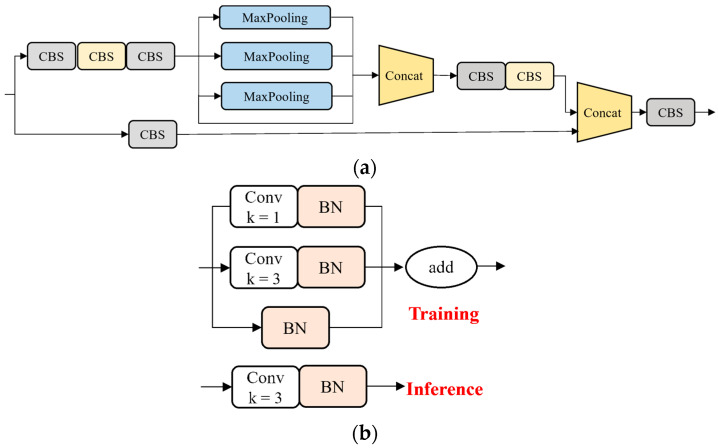
The structure of SPPCSPC and REP. (**a**) The SPPCSPC module. (**b**) The REP module.

The detection head receives three feature maps generated by the first, second, and third stages of the feature extraction network. These feature maps undergo a fusion process that combines fine-grained information with semantic information in a top-down and bottom-up manner, enhancing the ability of the network to perceive detailed information. Figure 5a illustrates the structure of the Spatial Pyramid Pooling and Cross-Stage-Partial-Connection (SPPCSPC) module, which consists of two branches. One branch combines different scales of maximum pooling and CBS to allow the network to adapt to images of varying resolutions. This branch is then concatenated with a CBS branch to expand the receptive field while minimizing the loss of high-dimensional feature information. The SPPCSPC module has two advantages: multi-scale feature extraction and efficient network design; the module first uses maxpooling of different sizes to capture information of different scales, and then combines the feature map with the original feature map to generate richer feature representations with multi-scale information. This approach not only helps the model better understand the objects in the image, but also maintains good performance at different resolutions.

The re-parameterized (REP) module [21] uses complex structures during training to enhance the expressiveness of the model, but re-parameterizes these complex structures into simpler forms during the inference phase to reduce computational overhead. It adopts a different structure, as depicted in Figure 5b. In the training phase, the REP module consists of Conv3×3, Conv1×1, and identity connections with BN. This multi-branch parallel structure enables the network to extract rich features. In the inference phase, the parallel structure is transformed into a Conv3×3 main branch. The REP indirectly reduces computational and parametric requirements, resulting in improved inference speed.

### 2.3. Loss Function

In object detection, the *IoU* is widely utilized to quantify the overlap ratio between the bounding box ($B_{pred}$) and the Ground Truth box ($B_{gt}$). The *IoU* is commonly defined as Equation (1):

(1) $$L_{IoU}=1-IoU(B_{pred},B_{gt})=1-\frac{Area(Intersection(B_{pred},B_{gt}))}{Area(Union(B_{pred},B_{gt}))}\in [0,1]$$

where $Area(Intersection(B_{pred},B_{gt}))$ represents the area of the intersection of $B_{pred}\;and\;B_{gt}$; $Area(Union(B_{pred},B_{gt}))$ represents the area of the union of $B_{pred}$ and $B_{gt}$.

**Figure 6:**
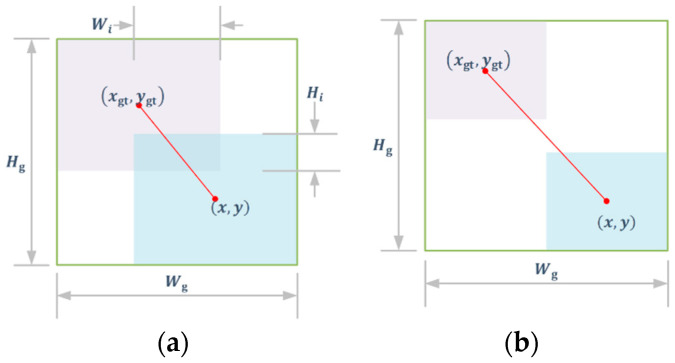
GTbox and Bbox position relationship. (**a**) Overlap. (**b**) No overlap.

When the Bbox and GTbox do not overlap, as in Figure 6b, the *IoU* lacks a gradient update, rendering it incapable of proper training. Additionally, to mitigate the influence of geometric factors (such as distance and aspect ratio) that may intensify the penalty on low-quality samples and consequently reduce the detection accuracy, a focusing mechanism based on the *WIoU* loss [22] is introduced.

The *WIoU* is defined as Equations (2) and (3):

(2) $$L_{WIoU}=\lambda \cdot R_{WIoU}\cdot L_{IoU}$$

(3) $$R_{WIoU}=\exp(\frac{{\left(x-x_{gt}\right)}^{2}+{\left(y-y_{gt}\right)}^{2}}{W_{g}^{2}+H_{g}^{2}})\in [1,e)$$

When the GTbox and Bbox have no overlap, as in Figure 6b, for *IoU*, $W_{i}$ and $H_{i}$ are 0, and $L_{IoU}$ is kept as 1. In this case, the *IoU* gradient stays frozen, unable to be utilized for training. For *WIoU*, λ and $R_{WIoU}$ are not simultaneously kept to be invariant, which means that $L_{WIoU}$ will not keep the 1 value; therefore, the gradient can be updated constantly. $W_{g}$ and $H_{g}$ are the width and height of the minimum closed box, as in Figure 6a.

The *IoU* is affected by the geometry factor between the Bbox and GTbox of low-quality samples, while *WIoU* can dynamically adjust the gain to reduce this impact. In *WIoU*, $L_{IoU}$ of low-quality Bbox can be amplified by $R_{WIoU}$, while $R_{WIoU}$ of high-quality Bbox is reduced by $L_{IoU}$. When the Bbox and GTbox overlap well, center point distance attention will be lowered.

(4) $$\lambda =\frac{\beta }{\delta \alpha^{\beta -\delta }},\;\beta =\frac{L_{IoU}}{\tilde{L_{IoU}}}$$

where β is the outlier degree of the Bbox, and a larger β means a low quality of the Bbox. λ is a non-monotonic focusing factor assigning small gradient gains to large outlier degree Bboxes, thus preventing large undesirable gradients from low-quality samples. $\tilde{L_{IoU}}$ is the running mean with a momentum of $1-\sqrt[{tn}]{0.05}$ (n is the batchsize value for training, and *t* is the number of epochs when the model’s AP boost becomes slow). Both the $\tilde{L_{IoU}}$ and bbox quality demarcation is dynamic, and therefore *WIoU* can dynamically assign gradient gain. After fine-tuning, the final hyperparameters α=1.9 and δ=3 are set.

## 3. Experiments and Results

### 3.1. Implementation Details

The experimental data are mainly collected by professional inspection vehicles using line array cameras for image acquisition on the surface of a highway. A dataset consisting of 1000 pavement-sealed crack images with a resolution of 640 × 640 was constructed. The datasets are first arranged out of order, and then randomly sampled into a training set, a validation set, and a test set, following an 8:1:1 ratio. The initial pre-training weights for the model were obtained using the VOC dataset [23], which were then utilized to facilitate transfer learning on the pavement-sealed crack distress dataset, preventing potential training degradation caused by excessive random initialization. To ensure experimental accuracy, a consistent training and testing environment was maintained throughout. The selected hardware acceleration device for training was an RTX2080 with CUDA 13.1. Data augmentation techniques including Mosaic and Mixup were employed with a trigger threshold of 0.5. Lastly, the hyperparameters were set as follows: an input size of (640, 640), optimizer as Stochastic Gradient Descent (SGD) with a momentum of 0.937, initialization learning rate of 0.01, weight decay of 0.0005, epoch of 300, and batch size of 16.

### 3.2. Ablation Experiments

**Figure 7:**
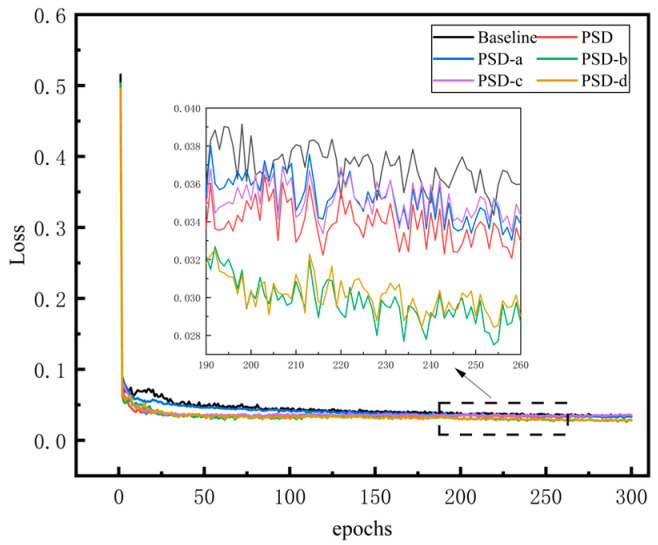
Loss update curve of ablation experiments.

To evaluate the influence of the PSD on detection performance, ablation experiments were conducted. These experiments involved adding SA modules, adjusting loss functions, and varying the scales of the detection head. The Baseline consisted of the IoU and three different scales of the detection head. Ablation experiments were conducted, ensuring that all models were sufficiently trained for fair comparisons. The loss curve during the experimental process is depicted in Figure 7. The training loss curve demonstrates an initial nearly linear decrease, with all models starting to converge around the 10th epoch. Subsequently, they continue to converge smoothly after approximately 200 epochs, reaching loss values below 0.04.

The evaluation criteria included mAP@0.5 (mean Average Precision with an *IoU* threshold of 0.5), Precision, and F1-score, as shown in Equations (5)–(7).

(5) $$Precision=\frac{TP}{TP+FP}$$

(6) $$Recall=\frac{TP}{TP+FN}$$

(7) $$F1=\frac{2\times Precision\times Recall}{Precision+Recall}$$

where True Positive (*TP*) refers to the number of samples correctly predicted as positive, False Positive (*FP*) indicates the number of samples incorrectly predicted as positive but are actually negative, and False Negative (*FN*) represents the number of samples incorrectly predicted as negative but are actually positive. Detailed experimental results are available in Table 1.

In Table 1, the structure of the Baseline is mainly composed of the model architecture introduced in Section 2, and its loss function is the IOU loss, which retains the output of the detection head at three scales. The SA module is not added in Stage 1 and Stage 2 of the feature extraction network. The number of detection heads is adjusted according to Figure 4. When the number of detection heads is 3, the output results from Stage 1 to Stage 3 are retained. When the number of detection heads is 2, only the output of Stage 1 and Stage 2 is retained, and the branching structure of Stage 3 is closed. The PSD-a was modified by replacing the detection head of the Baseline model with a two-way detection head specifically designed for pavement-sealed crack distress. The PSD-b was obtained by replacing the loss function of the Baseline with the WIOU. Similarly, the PSD-c replaced the loss function of the PSD-a with the WIOU loss function. Additionally, the PSD-d was obtained by incorporating the SA module into the PSD-b. Finally, the PSD was generated by adding the SA module to the PSD-c.

The metrics employed in the ablation experiment indicate that mAP@0.5 emphasizes positional accuracy, while the F1-score represents a composite measure of Recall and Precision. Consequently, this ablation experiment aims to compare the outcomes of these two indicators to identify the model exhibiting superior performance. According to Table 1, the Baseline model achieved an mAP@0.5 of 94.44% and an F1-score of 92.79%. Compared to the Baseline, the PSD-a model demonstrated enhancements of 1.87% in mAP@0.5 and 2.74% in the F1-score. The PSD-b model recorded improvements of 0.68% in mAP@0.5 and 1.25% in the F1-score, while the PSD-d model exhibited gains of 2.43% in mAP@0.5 and 1.67% in the F1-score. Additionally, the PSD model attained an mAP@0.5 of 98.02% and an F1-score of 0.9680, showcasing significant advantages over the Baseline and PSD-a models, which experienced losses in Intersection over Union (IoU). When WIoU is utilized as the loss function, the PSD model demonstrated enhancements of 2.90% in mAP@0.5 and 2.76% in the F1-score compared to the PSD-b model. Similarly, relative to the PSD-c model, the PSD model exhibited improvements of 0.65% in mAP@0.5 and 0.30% in the F1-score. When compared to the PSD-b model, the PSD model reflected improvements of 1.15% in mAP@0.5 and 2.34% in the F1-score. Further, the CIoU was used for ablation versus WIoU on PSD. The results showed that the PSD with CIoU as IoU loss was lower than WIoU in terms of mAP@0.5, Precision, and F1-score, respectively. Based on these ablation experimental results, the PSD model proves to be more effective in detecting pavement-sealed crack distresses than other structural models.

### 3.3. Experimental Results and Analysis

Table 2 presents the results of the comparison experiments conducted between the PSD and other representative one-stage detection models that are state-of-the-art (SOTA). To ensure experiment rigor, the parameters of the selected models were adjusted to align with the characteristics of the PSD.

**Figure 8:**
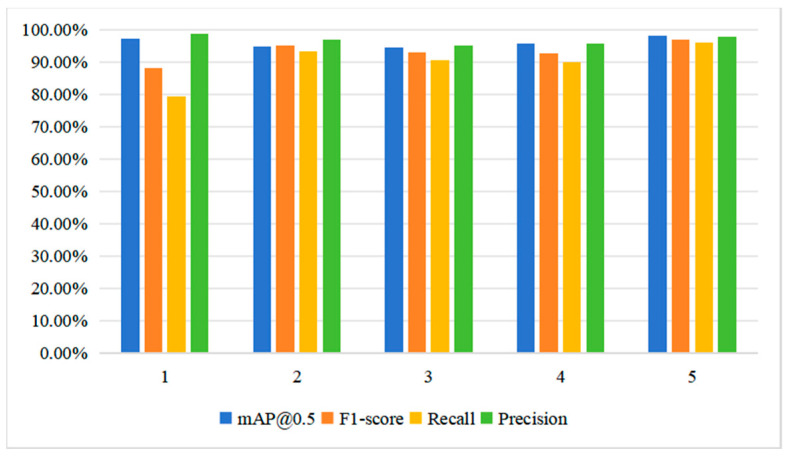
Comparisons with the state-of-the-art one-stage models.

**Figure 9:**
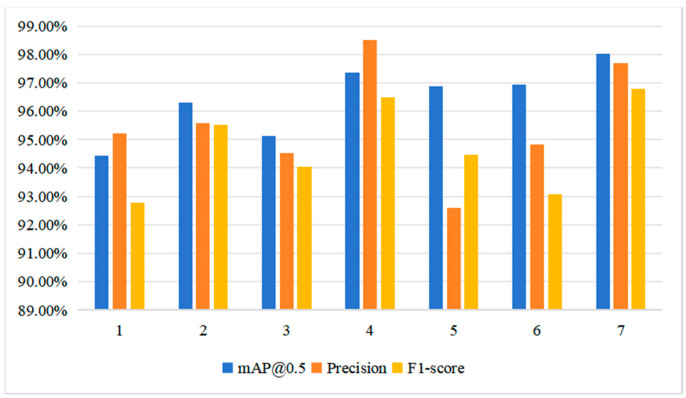
Comparison of ablation experiment results.

From Table 2 and Figure 8, the Precision, Recall, and mAP@0.5 of the PSD achieved 98.02%, 0.9680, and 0.9593, respectively. These results were 0.81%, 1.8%, and 2.79% higher than the best results obtained by the SOTA methods. While the PSD did not show an advantage in Precision compared to YOLOV5, it demonstrated a 16.52% improvement in Recall compared to YOLOV5. Therefore, the comparison results indicate that the PSD exhibits certain superiority in terms of Precision and Recall. To validate the effectiveness of the PSD, a random sample of four untrained images was selected for prediction. The inference results are illustrated in Figure 9.

**Figure 10:**
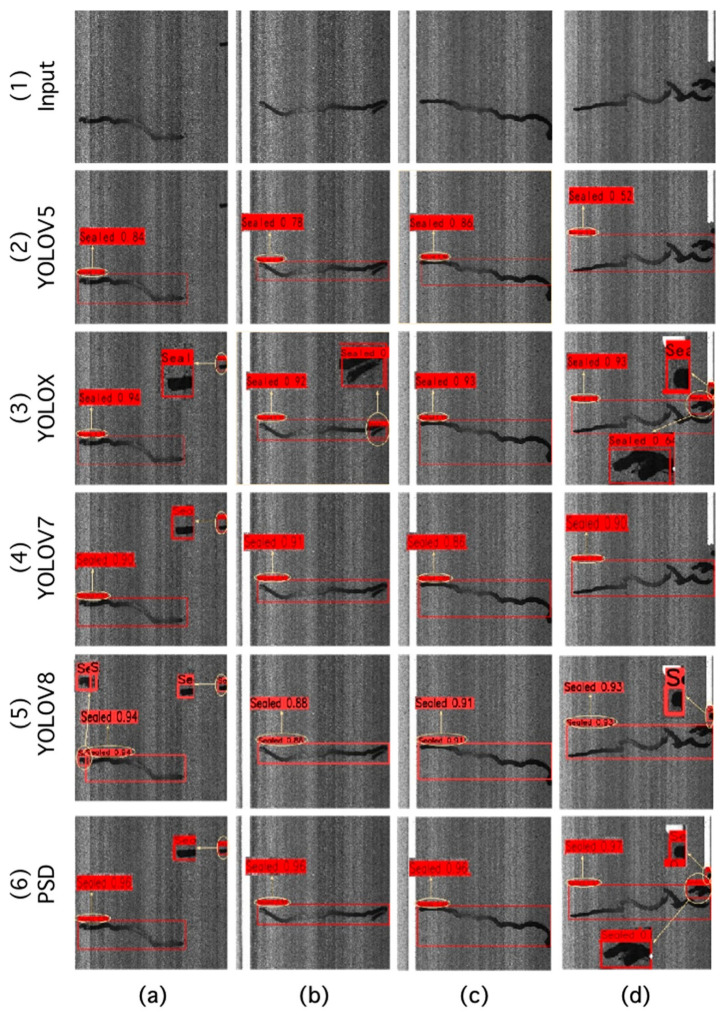
The visualization of results for different detection methods.

The first row of Figure 10 presents the original image of the pavement-sealed cracks, where (a) to (d) are random samples in the test set, respectively. Missed detections are observed in Figure 10 (a2,d2,d4,d5), while false detections occur in (a3) and (b3). Inaccurate localization is noted in (c2), (c4), and (c5). Notably, the PSD achieves the highest confidence score. For example, the confidence score of Figure 10 (b6) is 0.96, surpassing (b2), (b3), (b4), and (b5) by 0.18, 0.04, 0.05, and 0.08, respectively. The PSD not only demonstrates accuracy but also shows potential for addressing the issues of missed detection, false detection, and inaccurate localization.

**Figure 11:**
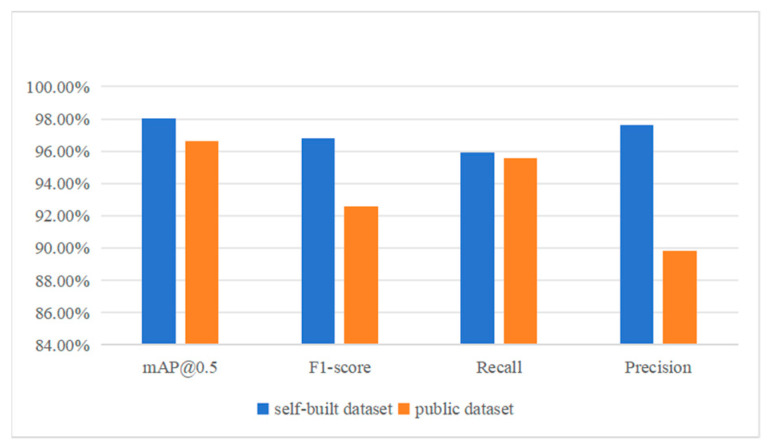
Comparison of PSD between self-built dataset and public dataset.

The above experiments prove that PSD has superiority in pavement-sealed crack detection on a self-built dataset compared with current SOTA object detection models, but in order to further verify the generalization performance of PSD, training and testing are carried out on a public dataset [18]. The comparison results are shown in Figure 11.

According to the comparison results in Figure 11, the mAP@0.5 of PSD on the public dataset reaches 96.6%, which is close to that of the self-built dataset, indicating that the positioning accuracy of PSD is stable under different data. The Precision and Recall were 0.898 and 0.956, respectively, and the F1-score was 0.926. The accuracy and F1-score are close to those of the self-built dataset. However, the Recall rate is slightly lower than that of the self-built dataset, which may be due to the large gap between the resolution of the public dataset and the self-built dataset; the original resolution of the self-built dataset is (1280, 1280), and the input size of the network after resizing is (640, 640), while the resolution of the public dataset is (600, 600), which needs to be stretched to achieve (640, 640), which may cause changes to the potting target. However, in terms of collation results, the performance of PSD in the public dataset is basically close to that of the self-built dataset, which indicates the generalization ability and migration of PSD.

## 4. Conclusions and Discussion

The proposed pavement-sealed crack detection (PSD) model has been designed to achieve the accurate and automated detection of sealed cracks in pavements. By integrating the spatial attention (SA) module into both the feature extraction network and the detection head, the model can selectively focus on critical features of sealed cracks while effectively extracting spatial and channel information. The detection head utilizes three feature maps produced by the feature extraction network, which are then processed through top-down and bottom-up multi-scale feature fusion using the Spatial Pyramid Pooling with CSP (SPPCSPC), Enhanced Large Kernel Attention Network (ELAN), and Multi-Path (MP) mechanisms. This fusion process enables the model to capture precise contextual information necessary for accurate detection.

Furthermore, the Weighted Intersection over Union (WIoU) loss function is employed to improve the localization accuracy of sealed cracks. Ablation studies have shown that the inclusion of the SA module and the WIoU loss function, along with the enhanced detection head, led to a significant 3.58% increase in mean Average Precision at 0.5 (mAP@0.5) and a 4.01% increase in F1-score compared to the Baseline model. These results confirm the efficacy of the proposed enhancements.

In comparative experiments, the PSD model achieved an mAP@0.5 of 98.02% and an F1-score of 0.9680, outperforming state-of-the-art methods by 0.81% and 1.8%, respectively. These findings indicate that the PSD model excels in sealed crack detection and offers a robust solution for this task.

For future research, we plan to broaden the experimental data to encompass various types of pavement distress, including cracks, potholes, and other defects, thereby enriching the dataset. Additionally, we aim to further optimize the model to enhance its overall performance and applicability across diverse pavement conditions.

## Figures and Tables

**Table 1:** Comparison of ablation experiment results.

No.	Method	IoU	Detection Head	SA	mAP@0.5	Precision	F1-Score
1	Baseline	IoU	3	×	94.44%	0.9521	0.9279
2	PSD-a	IoU	2	×	96.31%	0.9559	0.9553
3	PSD-b	WIoU	3	×	95.12%	0.9452	0.9404
4	PSD-c	WIoU	2	×	97.37%	**0.9852**	0.9650
5	PSD-d	WIoU	3	√	96.87%	0.9261	0.9446
6	PSD	CIoU	2	√	96.93%	0.9483	0.9307
7	PSD	WIoU	2	√	**98.02%**	0.9768	**0.9680**

**Table 2:** Comparisons with the State-of-the-art One-Stage Models.

No.	Method	mAP@0.5	F1-Score	Recall	Precision
1	YOLOV5	97.21%	0.8804	0.7941	0.9878
2	YOLOX	94.74%	0.95	0.9314	0.9694
3	YOLOV7	94.44%	0.9280	0.9050	0.9521
4	YOLOV8	95.70%	0.9271	0.8990	0.9570
5	PSD	98.02%	0.9680	0.9593	0.9768

## Data Availability

As this study involves multiple research teams, data is extremely valuable. Therefore, some or all data, models, or codes that support the findings of this study are available from the corresponding author upon reasonable request.
